# Community-based interventions to enhance healthy aging in disadvantaged areas: perceptions of older adults and health care professionals

**DOI:** 10.1186/s12913-018-3855-6

**Published:** 2019-01-05

**Authors:** Abirami Srivarathan, Andrea Nedergaard Jensen, Maria Kristiansen

**Affiliations:** Center for Healthy Aging & Department of Public Health, Faculty of Health and Medical Sciences, University of Copenhagen, Øster Farimagsgade 5, 1014 Copenhagen K, Denmark

**Keywords:** Aging, Health promotion, Interventions, Community, Europe

## Abstract

**Background:**

The number of older adults with different ethnic and socioeconomic background is steadily increasing. There is a need for community-based health promotion interventions for older adults that are responsive to ethnic and socioeconomic diversity among target populations. The aim of this study is to explore encounters between older adults living in disadvantaged areas and health care professionals in the context of community-based health promotion.

**Methods:**

Qualitative methods were used involving interviews and focus groups with older adults (*n* = 22) and municipal health care professionals (*n* = 8), and multiple observations were conducted. Data were analyzed thematically.

**Results:**

Findings show a gap between health promotion services and older adults due to a perception of services as being neither accessible nor acceptable in the context of complex health and psychosocial needs. Health care professionals reported trust, proximity and presence as fundamental factors for improving acceptability and accessibility of health promotion services.

**Conclusions:**

There is a need to develop participatory approaches to engage older adults who live in disadvantaged areas in municipal health promotion services and to ensure that these services are relevant to these groups.

## Introduction

An aging population is significantly changing the demographic landscape of Western societies [1]. The increasing number of older adults with different ethnic and socioeconomic backgrounds in communities across Europe and North America underscores the need for rethinking health promotion in order to ensure acceptability of and accessibility to social and health care services for diverse groups of older adults [2, 3].

Studies have demonstrated that there is widespread social and ethnic inequality in the acceptability to and accessibility of social and health care services for older adults [3, 4]. These inequalities are due to a complex interplay of individual and contextual factors that shape encounters between older adults and health care professionals [5, 6], including health conditions, psychosocial resources, language proficiency and communication skills [7, 8]. This interplay should be seen in an organizational context, where insufficient interdisciplinarity and lack of financial resources hinder optimal health promotion [9].

In this multi-perspective intervention study, we explore ways to adapt health promotion services to older adults by using the case of preventive home visits in Denmark. Preventive home visits aim to promote health and prevent the decline of functional ability by a systematic and individually focused dialogue with older adults who are not in contact with health care services in municipalities [10]. Themes discussed in conversations between older adults and professionals differ according to the needs and wishes of the particular person, but often encompass diet, alcohol, smoking, exercise, sleep, stress, unintentional loneliness and quality of life. Based on an assessment of needs, strategies are discussed often focusing on locally available activities and/or services that may help overcome unmet needs. Relatives may be present during the conversation. The uptake of preventive home visits in Denmark differs among different subgroups of older adults, which prompted a recent change in the law. Municipalities are now obliged to offer preventive home visits to older adults between 65 to 74 years who are in particular risk of decline in social, mental and physical functional capacity. Housing conditions, socioeconomic status, cultural background, civil status, language proficiency, morbidity patterns and polypharmacy are criteria used to identify older adults in need of a preventive home visit. The change in the law intends to ensure that the promotion of health for older adults is responsive to the needs of diverse groups, including ethnic minorities and more socioeconomically vulnerable individuals, who often underutilize community-based health promotion services. The goal is to ensure effective and innovative methods in the recruitment of older adults and delivery of services [4, 11].

## Methods

### Aim

The aim of this multi-perspective study is to explore encounters between older adults living in disadvantaged areas and health care professionals in the context of community-based health promotion using the case of preventive home visits.

The intervention study was conducted in collaboration between researchers, a Danish suburban municipality and a housing association. The content of the intervention is to involve local community members in recruitment to preventive home visits that are then delivered either in private homes or in community associations in the area by municipal health care professionals. Additional adaptation of the intervention focus on language barriers that are overcome through involvement of translators and translation of leaflets on preventive home visits and other material on municipal health promotion services, and courses in cultural competencies for municipal staff involved in the intervention. Two courses were arranged about diversity and sensitivity in providing health promotion in disadvantaged areas for municipal health care professionals and community volunteers in the intervention. Furthermore, health care professionals established information meetings about the adapted version of preventive home visits in a local resident café for older adults in the area. The results presented in this article focus on the complexity of encounters between health care professionals and older adults that shape the participation in health promotion services.

### Study setting

This study took place from September 2016 to April 2017 in a disadvantaged area in a suburb of Copenhagen, Denmark, comprised of 915 apartments with 2626 inhabitants. The housing area was built in the 1970s, is densely populated, and appears physically segregated from the surrounding communities. Relative to other disadvantaged areas in Denmark, socioeconomic vulnerability is prominent in this area, with only 29% of residents having more than a primary school education. A total of 43% of the population is unemployed, which is markedly greater compared to the municipality in general and has the inevitable result of low average household income. Furthermore, there is great ethnic diversity; 50 different nationalities are represented in this area. In terms of baseline status for preventive home visits for older adults in this particular area, only one preventive home visit was delivered in the period from January 2016 to August 2016, which indicates a need to seek and implement innovative ways to recruit for and deliver this service.

### Selection of participants

The inclusion criteria for participation in the intervention study were based on; 1) age (65 and above) in order to capture participants in the target group for the preventive home visits; 2) country of birth (including only older adults born in Denmark, Pakistan or Turkey) as these are the largest nationality groups in the area, and to balance the number of languages represented with the resources available for the study); and 3) place of residence. Participants were recruited during a range of different formal and informal activities in the housing area. This included health promotion activities arranged by the municipality as well as informal gatherings and individual encounters with potential participants. During recruitment, we collaborated with community leaders such as chairpersons of local mosques, volunteers and employees of the housing association and municipal health care professionals who are in close proximity to the area. Snowball sampling was also used. Researchers were present in the area at different times of the day, and had an office centrally located to ensure engagement with potential participants and to gain insight into contextual factors that frame aging and health behaviors.

The recruitment strategy including the use of gatekeepers enabled a more trusting relationship between interviewers and participants. The recruitment process was ongoing during the study period until saturation of data was achieved [12]. Due to snowballing, six participants were a bit younger than the age set out in the inclusion criteria, and five lived outside the studied disadvantaged area in demographically similar neighborhoods. We decided to include these participants in the interviews for ethical reasons as it was deemed problematic to reject their wish for contributing that appeared to be clearly driven by a perceived need for voicing their perspectives on living in this disadvantaged area. During analysis, no differences appeared in perspectives between the participants based on age or place of living. The perspectives of the participants who did not meet the inclusion criteria did not differ from the other participants.

The final sample of interviewees consisted of 22 older adults born in Denmark (6), Pakistan (5) and Turkey (11) with Danish, Urdu and Turkish as the majority languages (Table 1) and eight health care professionals, who are health promotion consultants working in close proximity to the area and planners, who are centrally located in offices at a distance from the disadvantaged area and involved in the planning and implementation of the intervention of preventive home visits (Table 2). All eight health care professionals participated in the provided courses focusing on cultural competencies. None of the older informants received the adapted preventive home visit during the study period, but a number of participants showed interest in receiving a visit. Most of the older adults were either retired or received social benefits, and had several chronical diseases such as hypertension, elevated cholesterol, non-insulin-dependent diabetes mellitus and rheumatism.

**Table 1:** Characteristics of participants (older adults)

Participant number	Age^1^	Sex	Country of birth	Years in Denmark^2^	Type of interview
1	2	Male	Pakistan	3	Individual
2	1	Male	Pakistan	2	Individual
3	3	Female	Turkey	2	Individual
4	1	Female	Turkey	1	Individual
5	1	Female	Pakistan	2	Individual
6	2	Female	Turkey	3	Individual
7	2	Male	Denmark		Individual
8	1	Female	Denmark		Individual
9	3	Female	Denmark		Individual
10	1	Male	Turkey	2	Focus group
11	1	Male	Turkey	3	Focus group
12	2	Male	Turkey	3	Focus group
13	2	Male	Turkey	3	Focus group
14	2	Male	Turkey	3	Focus group
15	2	Male	Turkey	3	Focus group
16	3	Male	Pakistan	3	Focus group
17	2	Female	Pakistan	3	Focus group
18	4	Male	Turkey	3	Focus group
19	3	Female	Turkey	3	Focus group
20	2	Female	Denmark		Focus group
21	2	Female	Denmark		Focus group
22	2	Female	Denmark		Focus group

^1^Age in years: 1 = [< 65], 2 = [65–74], 3 = [75–84], 4 = [85≤]

^2^Duration of residence in Denmark in years: 1 = [0–19], 2 = [20–39], 3 = [40≤]

**Table 2:** Characteristics of participants (health care professionals)

Participant number	Proximity^a^	Type of interview
23	Close	Individual
24	Close	Individual
25	Close	Individual
26	Centrally located at an office	Individual
27	Centrally located at an office	Focus group
28	Centrally located at an office	Focus group
29	Close	Focus group
30	Centrally located at an office	Focus group

^a^The degree of proximity to the everyday life of older adults living in disadvantaged areas

### Data collection

Data was collected through a combination of 13 individual semi-structured interviews, six focus group discussions and observations. The combination of individual interviews and focus groups enabled us to gain insight into both sensitive and intimate perceptions, and more dynamic discussion by participants of their experiences accepting and accessing health promotion services [13]. Each focus group involved two to six participants, and interviews lasted between 37 to 93 min. Interviews were conducted in a setting based on the participant’s own choice, with seven of the interviews conducted in the homes of the participants, one in a mosque and five in local community associations. Five of the six interviews with health care professionals were conducted at their place of work and one at the university campus in Copenhagen. Topics covered in the interview guide are presented in Table 3. The observations among older adults focused on physical structures, social interactions and relations, well-being and health behavior in everyday life [13]. Observations among health care professionals focused on their encounters with older adults living in the areas and the challenges that occurred in these encounters. All observations took place in different local settings such as diabetes workshops and Danish language classes.

**Table 3:** Topics explored in interviews with older adults and health care professionals

Interviews with older adults	Interviews with health care professionals
• Background information • Understandings of health • Perceptions of aging • Experiences with illness, the municipality and the Danish health care system • Knowledge, usage and satisfaction of health promotion services with emphasis on preventive home visits	• Background information • Implementing health services in disadvantaged areas • Encounters with target group • Organizational structures in the municipality: resources and competencies

### Data analysis procedures

All interviews were transcribed verbatim, and data were analyzed thematically informed by access to health care [14]. According to RM Andersen [14] access is defined by the potential and realized entry to services. Through multiple close readings of the material, we became familiar with the data and developed an impression of the text as a whole. The analysis was driven by themes emerging as important for the experiences of encounters between older adults and health care professionals that shape the usage of health promotion services in a municipal context. Data from transcribed interviews and field notes were coded into meaningful units. Finally, descriptions and concepts were summarized into main findings [15]. During our analysis, we explored similarities and differences in perspectives on acceptability and accessibility among older adults on the one hand and health care professionals on the other. Throughout the analysis process there was continuous discussion within the multidisciplinary research group to ensure intercoder reliability [16].

### Ethical considerations

Participants were encouraged to contact the interviewer after the interview to express any doubts and seek clarification. In some situations, the interviewer helped participants navigate relevant social and health care services in cases where unmet needs were identified.

## Results

In the following presentation of results, the main themes are presented with an analytic focus on the acceptability and accessibility of health promotion services. Overall, these concepts were shaped by complex health and psychosocial needs of the older adults in the context of everyday life.

### Perceptions of older adults: The relevance of health promotion services

In general, participants perceived themselves as healthy, and emphasized the importance of a healthy diet and mild physical activities on a daily basis. However, as the interviews unfolded, it appeared that most participants felt lonely, had multiple chronic diseases and a previous record of substance abuse. Also, most participants had financial strains that affected daily life, including their inability to pay medical bills and to participate in health promotion activities such as a membership to a fitness center. These challenges were also present during observations, as this was a common topic among the older adults. In light of the challenges, health was not given the highest priority. The participants perceived their chronic diseases as an inevitable part of aging processes and perceived health promotion services to be irrelevant. In one participant’s words,

“When you grow old, the thing is that you get sick, and you are not able to do the things you did before. I have accepted all of this from now on […] Now, if I take a walk or something, I easily get sick. But that is normal as well. Very normal, as I am so old.” (Participant 6).

Besides viewing functional decline as an inevitable part of aging processes, participants also expressed a need to be independent as a reason for not accepting health promotion services offered by the municipality,

“Well, I have heard, they [the municipality] have many services. But I do not feel like this is something I need, right. I mean, when you can take care of yourself, then why burden the municipality with a whole lot of things? This is my way of thinking.” (Participant 9).

This non-acceptance caused a lack of participation in available health promotion services such as preventive home visits. The participants’ complex health and psychosocial needs evolved around specific everyday challenges; factors such as financial strains, psychosocial issues and the perception of irreversible functional decline were of higher priority than active engagement in health promotion services.

### Encounters with the municipality: Limited acceptability and accessibility

Acceptability of health services among the participants was based on their previous negative encounters with social services. Participants perceived it difficult to distinguish and navigate the different services provided by the municipality; many viewed the various services as coming from one entity. As one participant described after being discharged from the hospital due to a blood clot,

“They [the municipality] came every day and monitored me. I said: I do not want them here anymore.” (Participant 7).

The municipality was often described as an authority that monitored citizens in their private homes. This description had become a common way for friends and family to speak about the municipality, which was further observed by the researchers in encounters with the older adults. In one participant’s words,

“I know a man, who went to the municipality. The municipal lady discussed way too much with him, asking questions such as: What are you doing? Why are you here? […] Hereby, there are many problems related to the municipality.” (Participant 16).

The negative perception of the municipality seemed to have a great influence on the participants’ encounters with it, and influenced their acceptance of health promotion services. However, when participants finally reached out to the municipality and expressed their need for concrete health promotion services, they experienced an inadequate response, and thus perceived services to be inaccessible. One participant complained about waiting for a long time for a response regarding rehabilitation from the municipality,

“I think that I have not received the help that I have asked for. So, for example three years ago, I got a new knee. And then I got approved for training to [training center] […] Now I have applied for the training again […] And I have not heard anything. I have not received any information [...] That is not all right, I think […] They can just bloody tell me, yes or no, instead of letting one wait and wait for an answer.” (Participant 8).

This view was shared by other participants, who had similar experiences,

“For instance I cannot polish my windows and such things. I actually asked for some help regarding that, but no, that is not delivered by the municipality.” (Participant 3).

During the interviews, participants expressed frustration and mistrust towards the municipality, and they experienced that their complex needs were not met. The needs of the participants were often very tangible; they had a practical rather than a preventive focus. When they received aids such as a walker, it was a one-time allowance that facilitated daily life, and did not necessarily promote health and prevent diseases.

### Perception of health care professionals: Proximity matters

The perceptions of the health care professionals on the lack of participation in available services were shaped by their proximity of their engagement in the everyday life of older adults. Some health care professionals expressed a need for trustful relationships, as they were familiar with the mistrust towards the municipality among older adults,

“I believe that it takes several years to become accepted in such [disadvantaged] areas […] You have to spend much time nursing these relationships, as there will always be some skepticism towards new people, like: Is he going to be gone in a year?” (Participant 24**).**

This negative view influenced the professional’s ability to approach the older adults. The skepticism was noticed during observations, where the older adults’ body language and attitude differed towards health care professionals in terms of their proximity. When engaging with the target group, the professionals were aware of the need for innovative ways of recruitment and delivery, with a focus on trust, proximity and presence in the encounter,

“They primarily come because of the social aspect [of health promotion services in the community], I think. For example, if you go to the residential café and talk with the residents, it is more a chit-chat, or if they are in pain or something, it [is] more that […] I think especially with these groups, where health is so unfamiliar to them […] You really have to begin by learning the alphabet, in which we do not talk about health at all. We have not come that far, yet.” (Participant 24).

The health care professionals reflected on whether they should be the primary contact to the older adults, and additionally the idea to use participatory approaches such as including local community members in preventive home visits. They referred to the complexity of psychosocial needs among the older adults. As one health care professional said,

“If we look at a hierarchy of needs, would it be the health issues that appear to be most important, or is it something else? Should it actually be other professions who made the first contact [to the older adults]? I think that one should make some considerations about that […]” (Participant 29).

Some health care professionals experienced an uncertainty in their encounters with the older adults, as they were perceived to be different in relation to language, culture and lifestyle. Health care professionals more centrally located described challenges engaging with diverse population groups, where factors such as ethnicity and social vulnerability were entangled with each other,

“They have other cultural behaviors, patterns of reactions and division of roles, which makes it more difficult for them to fit in the frame we have imposed. So, there are even more challenges, more parameters that are challenging.” (Participant 30).

In some situations, the lack of encounters between older adults and health care professionals were explained by social vulnerability and cultural diversity. In one health care professional’s words,

“[…] But they just would not receive a visit from us: That is not our business. I simply believe that is how it is […] We do catch some, but many are like this: Of course, that is not our business.” (Participant 28).

A difference emerged between the centrally located health care professionals and the ones in closer proximity to the disadvantaged area. The importance of trust, proximity and presence in health promotion services expressed by the older adults was shared by professionals in close proximity to the everyday life of the older adults. By contrast, professionals more centrally located described the lack of participation in available services as a result of communication difficulties, cultural diversity, social vulnerability and lack of acceptance of health promotion services.

## Discussion

In this article, we have explored encounters between older adults living in a disadvantaged area and health care professionals in the context of a targeted community-based intervention aimed at increasing the acceptability and accessibility of health promotion services. We have identified a gap between the provided services and the needs of the older adults that were reflected in a lack of participation in available services. This was partly due to a perception among the older adults of their functional decline as an inevitable part of aging processes, and partly due to a perception that services were neither accessible nor acceptable in the context of complex health and psychosocial needs. In addition, prior negative experiences with municipal agencies developed over the life-course of an individual added to the perceived unacceptability of health promotion services provided by the municipality. Health care professionals working in the particular area emphasized the importance of trust, proximity and presence as fundamental to improved acceptability and accessibility of health promotion services. This finding corresponds with A O’Mara-Eves, G Brunton, S Oliver, J Kavanagh, F Jamal and J Thomas [11], who emphasizes a need for innovative approaches to ensure that services are relevant to groups that are currently not participating.

### The gap between what is needed and what is provided

Health promotion in disadvantaged communities may be challenging [17]. However, this study stresses a general willingness to offer health promotion services to older adults living in disadvantaged areas. In accordance with our findings other studies have found that older adults living in disadvantaged areas have complex health and psychosocial needs [18, 19]. However, the municipality often tends to focus on individual behavior centered on lifestyle. The problem with focusing on individual behavior is that it does not recognize the context of complex health and psychosocial needs of this target group. Hereby, a gap between the provided services and the actual needs of the older adults occurs, highlighting for the need for a rethinking of health promotion services in disadvantaged areas. This finding is supported by a study by J Naaldenberg, L Vaandrager, M Koelen and C Leeuwis [20], who identified a discrepancy between the everyday life of the older adults and the health promotion services provided. The services offered by the municipalities are often based on specific health behaviors that many older adults find difficult to incorporate in the everyday life. The health care professionals in our study also noticed this gap. It is noteworthy that the gap was perceived differently among the professionals and that the proximity of engagement with the older adults seemed to be an important factor shaping perceptions. This suggests that the closer the proximity to the target population, the more acknowledgment of the significance of including psychosocial aspects into health promotion by health care professionals. Our study therefore suggests that health should be assessed in a holistic manner for older adults living in disadvantaged areas, and health promotion services should accommodate this complexity [18].

### Lack of acceptance of health promotion services caused by mistrust

During interviews, the majority of the older adults expressed mistrust of the municipality. Consequently, the mistrust increases the risk for a suboptimal encounter or at worst no encounter at all between the older adults and the health care professionals. R Suphanchaimat, K Kantamaturapoj, W Putthasri and P Prakongsai [21] found that mistrust of health care providers was a major barrier for access to health care among migrants, and studies [4, 22] have shown that migrants may hold negative perceptions of accessibility and quality of health care based on language barriers, differences in expectations and perceived discrimination. These studies correspond well with our findings in that health care professionals in our study perceived higher levels of mistrust among the older adults toward the municipality, independent of ethnic identity. This awareness caused uncertainty in their encounters with the older adults, which enhanced the existing gap, as the professionals were hesitant to engage in encounters. Furthermore, professionals expressed a fear of being insulting and insensitive in encounters with older adults, and found it difficult to assess their health promoting needs. This suggests that cultural competencies should be enhanced among professionals and that increased skills and competencies of the individual should be supported by organizational changes to allow for more time to build trusting relationships across ethnic and socioeconomic characteristics [23, 24].

### Providing accessible and acceptable services by engaging community members

Our study indicates a need for participatory approaches in the recruitment and delivery of health promotion services in order to ensure acceptability and accessibility of these services. Involvement of community health workers through health fairs and out-reach activities hold potential to improve delivery of health promotion services in underserved communities [3, 25]. There is an unexplored potential of preventive home visits based on participatory approaches.

In line with our study A O’Mara-Eves, G Brunton, S Oliver, J Kavanagh, F Jamal and J Thomas [11] indicates that a collaboration between the municipality and members of the local community could be beneficial. The importance of community engagement, trustful relationships, relatable professionals and appropriate delivery of messages and materials is emphasized in study by JJ Liu, E Davidson, R Bhopal, M White, M Johnson, G Netto and A Sheikh [26] focusing on health promotion interventions in ethnic diverse populations.

### Methodological considerations

As our findings are situated in a particular context, it is not our intention to generalize to other groups and contexts. However, we expect to find some of the same gaps between health care professionals and older adults with complex needs living in similar disadvantaged areas.

The present study is explorative and further research in the field of older adults’ accessibility to and acceptability of community-based health promotion services should be initiated. Factors such as ethnicity, migration history and gender differed among participants, and naturally, this resulted in diverse life stories.

Despite the diversity of the study participants, common characteristics among the participants were found. All participants were older adults at risk of reduced social, mental and physical functional capacity in accordance with the target group of preventive home visits. Furthermore, all participants live in an area that is often described negatively by politicians and the media as socially disadvantaged.

The use of interpreters during six of the interviews challenged the interaction between interviewer and participants [27]. However, the interpreters were informed of research ethics and guidelines for interpretation prior to the interviews, and in general facilitated trustful and nuanced interactions. The validity of the study was strengthened by analyzing the data individually, followed by comparisons of emerging themes, and by continuously presenting findings to the multi-disciplinary research group.

### Implications for policy, practice and research

A continued policy focus on enhancing equality in healthy aging across diverse groups of older adults, including those living in disadvantaged areas, is needed. Our study indicates the need for early interventions in the life-course of the individuals both to ensure timely and relevant health promotion services and to bridge the perceived gap between more marginalized groups and service providers. Policies should acknowledge that health promotion services in disadvantaged areas may be resource-demanding, as both recruitment and delivery are more time-consuming if a trustful relationship between health care professionals and older adults is to be established. Furthermore, health promotion services, including preventive home visits for older adults living in disadvantaged areas, should consider the psychosocial factors of health to a greater extent. This calls for cross-disciplinary teams and collaboration across health and social care services.

In terms of practice development, more focus on staff competencies in engaging ethnic and socioeconomic groups in community-based health promotion is needed.

This study has a multi-perspective approach on the encounter between older adults and health care professionals. However, quantitative methods such as surveys and register studies are needed to describe access to and the quality of health promotion services for diverse groups of older adults in different communities and across countries. This will enable identification of groups in greater need of targeted services and dissemination of good practices across settings.

## Conclusions

This study has explored the acceptability and accessibility of health promotion services among older adults living in a disadvantaged area. The findings point to a number of barriers that prevent older adults from using health promotion services. The findings also highlight the need for participatory approaches to engage older adults living in disadvantaged areas and to ensure that services encompass the complex health and psychosocial needs of these older adults.
